# From honest mistakes to fake news – approaches to correcting the scientific literature

**DOI:** 10.1186/s13005-020-00220-8

**Published:** 2020-04-03

**Authors:** Thomas Stamm

**Affiliations:** Department Orthodontics, University of Münster, Waldeyer Str. 30, Münster, 48149 Germany

## The dilemma

On the occasion of recently published errata in Head & Face Medicine [1–3], the question arose how to deal with errors in a published article. Making, discovering and correcting errors is a normal process in science and is often referred to as a self-correcting process. Fortunately the number of errata in Head & Face Medicine is very low [1–5] and so does not pose a risk in making clinical decisions.

Finding an error in a scientific publication is one thing, correcting it is a totally different world. *"Science journals have the luxury of time"* [6]. This quote highlights the advantage of having enough time to carefully review and correct an article prior to publication. It also draws attention to the disadvantage that making changes to an article once published is quite difficult. Publications contaminated by errors coexist with their healthy counterparts in different databases, and in the worst case scenario, can multiply in systematic reviews and meta-analyses. The mass production of such summarizing data collections contributes significantly to the dissemination and survival of false data. Garmenia and coworkers [7] found that 46% of all re-assessed meta-analyses would result in a change in the initial findings if studies with falsified data were to be excluded. The authors concluded that *"Falsified data can affect not only the original publication, but also any subsequent meta-analyses and any resulting clinical or policy changes resulting from the findings of these studies."* [7].

## The letter to the editor

What options are there for corrective intervention? Ethical scientists have a range of possibilities (Table 1). But, what about dealing with suspected misconduct? Is *"Letters to the Editor"* still an effective mean for correcting or retracting a false study? Probably not, as some illustrative examples have shown [8–10]. It is not recommended to contact editors and/or authors for potentially problematic work [10]. Oransky and Marcus explained that *"Contacting authors before anyone else knows about potential issues in their work, only serves to give unethical scientists time to hide their tracks – and let’s face it, those who are actually guilty of misconduct probably don’t have any scruples about covering up the evidence of that misconduct."* and *"While we’d like to be able to say that we find all journal editors responsive to allegations, there are still too many who rebuff efforts to correct the literature,* [10].

**Table 1:** Retraction guidelines and definitions adopted from the Glossary of retractions [11], Committee on Publication Ethics [12], and International Committee of Medical Journal Editors [13]

**Group**	**Action**	**Definition**
		Requesting concerns to
		-Institutions of authors
Editors	Correspondence	-Institutions of co-authors
		-Person responsible for research governance
		-Regulatory body
Editors, authors, readers	Letters	Published letter to the editor
Editors	Editor’s note	A minor point issued by the editor.
Editors	Editor’s warning	An issued statement by journal editors eliciting concern over the validity of a given paper or study.
Editors	Expression of concern	A statement issued by the editor to question the validity of a paper or portions of that paper.
Editors, authors	Errata	Published corrections
Editors, authors	Partial retraction	Retraction of a portion of a paper
Authors	Retraction	Formal withdrawal of one or more papers by one or all of the authors
Authors, editors	Retraction with republication	Replacement in case where a honest error (e.g. miscalculation) leads to a major change of the results of the original paper
Editors, institutions, funders	Retraction without permission	The formal withdrawal of one or more papers by a journal editor, the institution where the study took place, one or more of the papers authors, or funders.

## Community guidelines

The scientific community provides some guidelines for dealing with potential problems in scientific work and it is possible to identify a number of different groups who may act: a) editors, b) authors, c) readers, d) institutions, and e) funders (Table 1). According to the glossary of retractions [11] editors have the greatest number of options in resolving potential issues. In the case of honest mistakes the principal aim should be to correct the work without harming the authors’ reputation. Authors themselves have the opportunity of correcting, partially retracting or withdrawing their work with a detailed explanation regarding the reasons. The only reasonable course of action in the case of evident misconduct is retraction without the authors’ permission. This can only be carried out by editors or institutions in which the work has been investigated.

## Retraction reasons

**Fig. 1:**
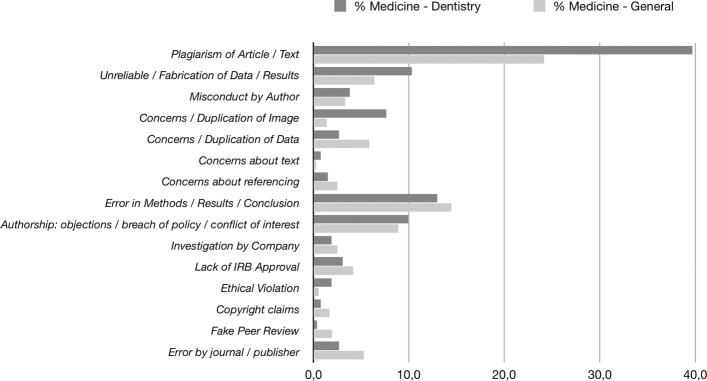
Decisions for retraction obtained from the Retraction Watch Database (http://retractiondatabase.org) for the subjects *"Medicine – Dentistry"* (n = 181 articles) and *"Medicine – General"* (n = 212 articles). Percentages of occurrence are shown

The reasons for retraction are different and have developed over time. For example, plagiarism is a more recent kind of offence [14]. Scientific misconduct is not limited to data fabrication: diverse forms of misconduct may lead to retraction. The Retraction Watch Database (http://retractiondatabase.org) provides several reasons for retraction. Carrying out a search under subjects *"Medicine – Dentistry"* and *"Medicine – General"* revealed 25 different decisions for retraction. Some of these overlap so that overall 15 reasons result (Fig. 1). Plagiarism is one of the most frequently occurring types of misconduct. Surprisingly, it occurs more frequently in dentistry than in medicine. The introduction of plagiarism-detection software may be the reason for its higher occurrence in both disciplines. Concern about methods and results, including data fabrication, is the second most frequent fraud. The third most frequent basis for retractions is authorship disputes.

## Editors’ options

It is indisputable that fabrication or falsifying data is the most dangerous fraud in medical science. It is essential that this category of misconduct must be countered resolutely at all levels. Until now there have been many recommendations and guidelines requiring editors and journals to respond appropriately to fraud [12, 13]. The Committee on Publication Ethics (COPE) provides several flowcharts on how to correspond and with whom in alleging misconduct [12]. The International Committee of Medical Journal Editors (ICMJE) has made aware the lack of value of a letter to the editor in some circumstances by stating *"Expressions of concern and retractions should not simply be a letter to the editor."* [13]. ICJME highlights the importance of proper indexing: *"Rather, they should be prominently labelled, appear on an electronic or numbered print page that is included in an electronic or a print Table of Contents to ensure proper indexing, and include in their heading the title of the original article."* [13].

These rules apply exclusively to editors and journals. In terms of the World Medical Association Declaration of Helsinki, not only editors and journals but every physician is ethically obliged to correct errors and combat fraud. But what if editors and journals are reluctant to respond to allegations? What can the individual do?

## What individuals can do

Social media is one tool for raising concerns about published work. Such allegations are already dealt with in the guidelines for editors [12]. Depending on the platform and reach, this can attract more attention than a letter to the editor which, indeed, may even never be published. Post-publication peer review (PPPR) on specially dedicated websites and blogs is a further process that is faster than traditional forms of evaluation. PPPR shifts discussions into the public domain online where discussions can be rapidly disseminated through and by any interested individual [15]. The effectiveness of platforms such as PubPeer (http://www.pubpeer.com) depends on the online skills of individual journal editors, whose attention is to be aroused. A good overview of PPPR platforms can be found in a paper by Paul Knoepfler [15].

Another option is to submit a response article that disproves the results of the paper being suspected of fraud. However, as is the case with letters to editors an increased risk remains that the disproving article may not be published. Knoepfler [15] describes a case of retraction where a rebuttal article was rejected but later published elsewhere.

Possibilities available for the scientific community to intervene in the process of correction are few. As already mentioned, there is a particular dilemma when false data are aggregated to new knowledge into a meta-analyses, because its existence will continue, even if included studies have been withdrawn. One solution might be a new, updated meta-analysis, if the withdrawal of studies has an impact on the initial findings. Moreover, in accordance with the ICMJE recommendations for proper indexing, the original title and keywords should be enclosed in the update.

## The rebuttal article

In order to avoid the slow and sometimes ineffective paths via journal mechanisms, intervention could take place directly during the search through a literature database. Once a study has been identified for meta-analysis, a written concern (if any) regarding the study should also appear in the search results. This can be achieved by publishing a rebuttal article or a critical assessment with nearly the same title and key words, to ensure equal indexing in electronic databases. Examples in this direction have been provided by The Journal of the American Dental Association’s JADA + Clinical Scans (JADA.ADA.org /ClinicalScans). This section aims to provide a scientific- and evidence-based assessment of research. The terms *"Insufficient evidence..."* [16], *"No trustworthy evidence..."* [17], *"Serious limitations..."* [18] and many more warnings were used with the original title of the work under consideration. Such assessments, that appear in searches of medical databases, may help physicians in selecting the appropriate study for research and for the treatment of patients.

## Conclusion

To support the self-correcting process of science Head & Face Medicine is open to constructive criticism. The journal editors welcome any critical debate about their published papers if it is scientifically sound and formulated in an ethical and objective manner.

## Data Availability

Upon request to the author.
